# Traditional and faith-based healthcare in the management of psychotic disorders in Africa: in search for synergy

**DOI:** 10.1097/YCO.0000000000000872

**Published:** 2023-05-17

**Authors:** Martine C.E. van der Zeijst, Wim Veling, Bonginkosi Chiliza, Hans W. Hoek

**Affiliations:** a Parnassia Psychiatric Institute, The Hague; b Department of Psychiatry, University Medical Center Groningen, University of Groningen, Groningen, The Netherlands; c Department of Psychiatry, Nelson R. Mandela School of Medicine, University of KwaZulu-Natal, Durban, South Africa; d Department of Epidemiology, Mailman School of Public Health, Columbia University, New York, New York, USA

**Keywords:** Africa, faith-based healthcare, mental health gap, psychosis, traditional health practitioner

## Abstract

This review summarizes the current literature on the role of traditional and faith-based healthcare in the management of psychotic disorders in Africa.

**Recent findings:**

In contemporary Africa, individuals with psychosis and traditional and faith healers (TFH) are pluralistic towards their understanding of psychosis and their help-seeking behaviour. Traditional healing is perceived to be helpful to patients with psychotic disorders and their family members and may have a positive influence on the course of psychosis in some selected individuals. Studies show that potentially harmful practices are commonly used by African TFH, but that these are associated with a lack of resources and are susceptible to training. Although various TFH and biomedical practitioners are open to collaboration, the many identified obstacles hinder actual partnerships. However, the few studies that have been conducted on collaborative care for patients with psychotic disorders on the continent, show positive outcomes.

**Summary:**

Rather than harmonizing the two healing paradigms, synergistic collaboration between traditional/faith-based and biomedical mental healthcare in the management of individuals with psychosis seems to be possible within certain limits. Synergistic collaboration is more culturally syntonic and may actually contribute to bridging the treatment gap for mental disorders in present-day Africa.

## INTRODUCTION

Although psychotic disorders have a relatively low worldwide prevalence of approximately 1% [1], schizophrenia has been identified as a global mental health priority [2]. Schizophrenia is one of the most burdensome illnesses worldwide [3], not only for affected individuals but also for their caregivers [4,5]. Furthermore, it is estimated that 69% of individuals with psychosis in the world are not receiving mental healthcare in biomedical settings, and that this is even 90% in sub-Saharan Africa (SSA) [6]. On the African continent, access to biomedical treatment for psychotic disorders is limited [5]. Compared with the rest of the world, the African region has the lowest numbers of mental health workers (0.9 per 100 000 population), outpatient care facilities (0.07 per 100 000 population) and mental health beds (2.0 per 100 000 population) [7]. Also, financial hardship and long distances to treatment locations contribute to the limited accessibility of psychiatric care [5].

The sociocultural context of people influences how they understand and experience mental illness, and where they seek care [8]. In SSA, individuals with psychotic disorders who do seek treatment, usually receive care from both traditional and faith healers (TFH, term explained below) and biomedical mental healthcare practitioners [5,9]. Most often, TFH are the first to be contacted for mental disturbances including psychosis [5,10,11^▪^,^12^]. Not only are TFH easily accessible, but they also have the same sociocultural understandings as their patients regarding the underlying causes and treatment of psychosis [13^▪^], which are perceived to be based on a complex three-dimensional interplay between the self, the social world and the spiritual world. In case of imbalances, TFH are trusted to be able to restore these [14]. Traditional methods of care are usually less associated with stigma than biomedical treatment [9,10].

The literature on effective interventions for individuals with psychotic disorders in Africa, either biomedical or traditional, is scarce [13^▪^,^15^–^17^]. A systematic review showed that antipsychotic drugs, known to be the mainstay of treatment of schizophrenia [18], seem to reduce psychiatric symptoms in African patients with primary psychotic disorders [15]. However, side effects, nonadherence and antipsychotic polypharmacy occur frequently [5,9,15,19], and the access to and availability of antipsychotics is very limited [5,20^▪^]. Also, other forms of psychiatric treatment are often discontinued due to perceived ineffectiveness, high costs and the experiences of insulting remarks by biomedical practitioners [21,22^▪▪^]. The WHO writes that ‘There is clear evidence that mental hospitals are not effective in providing the care that people with mental health conditions need and, regularly, violate the basic human rights of persons with schizophrenia’ [23].

Many studies have shown that TFH are playing a role in the treatment of mental illness in Africa [5,10,11^▪^,^24^]. There are also indications that traditional treatment might improve symptoms in patients with psychosis [13^▪^,^17^,25^▪^,^26^], maybe especially during the acute phase of schizophrenia [17,26]. However, the (biomedical) evidence is sparce and the quality of the research evidence is rated as low [13^▪^,^17^]. Nevertheless, the majority of patients with schizophrenia who visit TFH, experience benefits of traditional treatment and the adherence rates may surpass 80% [13^▪^,^27^,^28^]. In addition, TFH are able to offer in-patient care at a much larger scale than biomedical mental healthcare facilities [29].

In order to scale up mental health services in Africa, the WHO has called for integration of traditional medicine into biomedical health systems for over 40 years [30]. In the meantime, most African governments have recognized the potential role of TFH in mental healthcare and scholars have long debated the merits of traditional health practices in the field of mental health [11^▪^,^17^,^28^,^31^]. Review papers [11^▪^,^32^] have shown that the traditional and biomedical healthcare systems are more compatible than previously thought in terms of illness causation and management of patients with mental health problems. For example, several African TFH not only recognize spiritual but also biopsychosocial causes of mental illness, use history taking, signs and symptoms for diagnoses and apply social and pharmacological interventions [11^▪^]. Yet, TFH are still not formally implemented in the African mental healthcare system [11^▪^,^31^,^32^]. One of the main barriers regularly mentioned in the literature, is the use of treatment practices by some TFH, which may be harmful to patients [31]. In the present review, we evaluate the value of TFH in the management of psychotic disorders in contemporary Africa. 

**Box 1:**
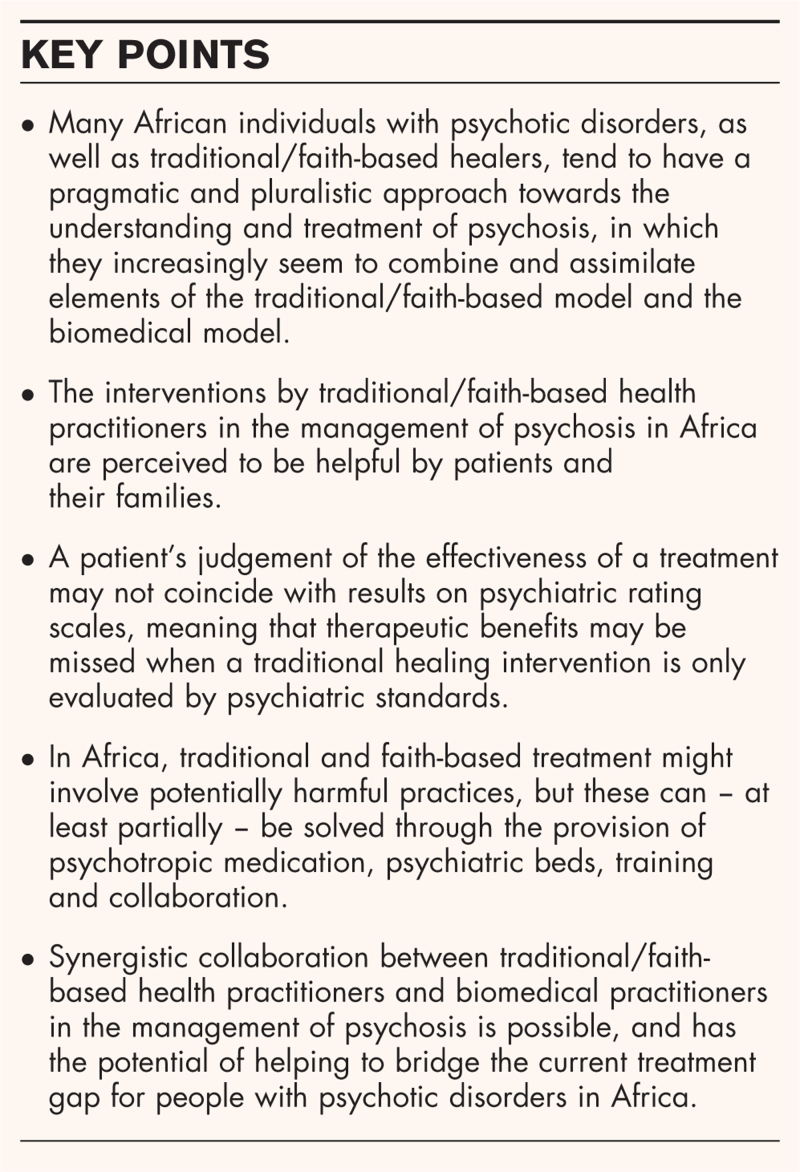
no caption available

## MATERIALS AND METHODS

A literature search was conducted in Medline and Pre-Medline, EMBASE and APA PsycINFO. We aimed to include recent articles at the intersection of the areas traditional healing, mental disorder and Africa, and used a search strategy with several synonyms and related terms for these topics. Studies which did not involve psychosis or any psychotic disorder or did not include traditional or faith healing were mostly excluded, except for some studies which provided important context. We supplemented the results with Google Scholar searches for specific combinations of terms and manually searched the reference lists of included articles. The search was conducted up to two January 2023 and limited to publications in English, Afrikaans, Dutch and German from 2020 onwards. The titles and abstracts of 478 unique listings were screened by the first author for relevance. We report in more detail on 18 articles in this narrative review. Search strategy is available upon request.

## RECENT AFRICAN STUDIES

The main themes investigated in recent African studies include causal models; help-seeking behaviour and pathways to care; practices and interventions; (perceived) effectiveness; harmful practices; and collaboration.

### Causal models

Two qualitative studies from Kenya, Nigeria, Ghana and Zimbabwe [22^▪▪^,^33^] showed that supernatural causal explanations of psychosis – mainly witchcraft, magic and ancestral and evil spiritual forces – remain predominant in all four countries. However, psychosocial causations such as ‘thinking too much’ and ‘stresses of life’, and biological causations such as head injuries, infectious diseases and familial predisposition were also reported at all sites, as were combinations within or between the supernatural, psychosocial and biomedical models [22^▪▪^,^33^]. Apart from the remarkable overlap in illness attributions between these African cultures, some differences were also found. For example, Ayinde *et al.*[22^▪▪^] found that psychosocial and biomedical causal explanations of psychosis were reported far more often by Kenyan patients than by Nigerian and Ghanaian patients. According to the authors, this may be a result of the investments that researchers in Kenya have made which were aimed at increasing knowledge of mental disorders among TFH and promoting active and ongoing dialogues between TFH and biomedical mental health practitioners [22^▪▪^]. The same authors also found that if patients with psychosis endorsed a natural causation, they often believed that they were more susceptible for a natural illness due to supernatural factors. As is the case globally, a study from South Africa [34^▪^] suggests that people are pragmatic towards the cause. That is, if a certain treatment is ineffective, other causations are considered. These findings imply that, in present-day Africa, there is a certain degree of fluidity, intermixing and assimilation within and between the three etiological categories of psychosis: supernatural, psychosocial and biological [22^▪▪^].

Two studies among TFH in South Africa [34^▪^,^35^] and one study among people from Burkina Faso who were identified as indigents with psychotic-like experiences [36], reported that, within the supernatural causal model, psychotic disturbances may be positively valuated as a supernatural gift of healing in some cases. For example, in a rural Zulu community, ‘the calling of the ancestors to become a traditional healer’, a cultural construct for psychotic disturbances, is regarded as a gift rather than an illness, despite the severe agony that individuals might experience at the onset of this ancestral calling. If in this rural Zulu community an individual presents him or herself with psychotic symptoms at a TFH, the TFH will evaluate whether these symptoms are caused by ancestral spirits, for example in the context of the positively valuated ancestral calling, or by evil spirits, which are associated with ‘madness’ [34^▪^].

### Help-seeking behaviour and pathways to care

A study from Egypt [21] showed that 42% of 232 patients with diagnosed schizophrenia had visited a TFH before consulting psychiatric care. Motives for initial consultation of a TFH were related to stigma (30%), costs (25%) and distance (16%). Ayinde *et al.*[22^▪▪^] found that the majority of 85 patients who had received treatment for psychosis at a TFH in Nigeria, Ghana or Kenya, had also consulted other practitioners, either concurrently or consecutively: another traditional healer in 36%, a Christian faith healer in 40%, an Islamic faith healer in 8% and a biomedical practitioner in 73%. The authors reported that a biomedical provider was attended especially when a patient suffered from accompanying somatic symptoms or perceived the cause of psychosis to be physical as well, and that patients regarded biomedical treatment as supplemental to traditional treatment [22^▪▪^]. This pluralistic help-seeking behaviour was also found in an ethnographic study from South Africa [34^▪^], and is consistent with most literature [5,10,11^▪^,^12^].

A study from Northern Malawi on pathways to care among 140 first episode psychosis patients [37] showed that referral from a TFH, as well as from a community-based volunteer, was four times more likely to be associated with a long duration of untreated psychosis (>6 months) than referral from a general practitioner. According to the authors, this was probably because many patients had been ‘admitted’ to the houses of the TFH up until the healers were asked to refer them to the study. The diagnosis of schizophrenia and unemployment was also associated with referral by a TFH or community-based volunteer. No relation was found between faith-based source of contact and psychiatric treatment delay [37], consistent with a previous study [38].

### Practices and interventions

Across TFH settings, treatment of patients with psychosis usually starts after a careful assessment, and includes a combination of herbal medications, rituals and prayers [22^▪▪^,34^▪^,^35^]. More specific, in Kenya, Nigeria and Ghana, the treatment performed by traditional healers and some Islamic faith healers may include animal sacrifice, oral herbal preparations, herbal snuff, a certain diet and music. The treatment performed by Christian faith healers may consist of holy water, spiritual baths, vigils, skin oil, fasting and music [22^▪▪^]. In these countries, prayers and faith were believed to heal evil causes of psychosis and give back to a patient what was lost during the illness [22^▪▪^]. The herbal medication made by Swati TFH from South Africa, consisting of a variety of plants and animal substances, which can be administered via oral ingestion, inhalation or bathing, is believed to heal psychotic disturbances by cleansing out evil intrusions [35]. In an exploratory ethnobotanical survey from Mali, Mounkoro *et al.*[39] collected the plant species, which TFH use in the management of local psychotic syndromes. On the basis of a literature study, the authors found evidence for interesting biological effects of some of the plants, such as inhibition of acetylcholinesterase. In addition, a recent review of a number of medicinal plants used as antipsychotics shows that some studies suggest antipsychotic potential of specific plants used in African traditional medicine [40].

Patients with psychotic disturbances are often ‘admitted’ in the homes of TFH until they show recovery [22^▪▪^,34^▪^,^35^]. In Ethiopia, individuals with severe mental illness, including psychotic disorders, may reside at holy water sites for a few months or multiple years [41^▪^,^42^^▪▪^].

In a series of qualitative and quantitative studies from rural KwaZulu-Natal, South Africa [25^▪^,34^▪^,^43^], a locally available intervention for individuals with psychotic disturbances was explored. This intervention is called ‘ukuthwasa’ in isiZulu, and can be translated as ‘the process of becoming a traditional healer’. During ukuthwasa, a selected individual resides with a traditional healer as an apprentice. The traditional healer helps the apprentice with the interpretation of the ancestral voices and the integration of the psychotic experiences into a cultural framework. Furthermore, the apprentice is prescribed traditional medication and is attending rituals, while being trained a new and respected role as a traditional healer [34^▪^].

### (Perceived) effectiveness

Whether a certain treatment is evaluated as effective, depends on how effectiveness is defined. Biomedical psychiatry evaluates the effectiveness of an intervention on the basis of symptom reduction as measured on a validated psychiatric rating scale. This approach focuses on quantitative changes, rather than on qualitative changes in meaning, perception of self, patient role and social interactions. Cultures vary significantly in how they conceptualize mental health and illness, and many attribute mental illness to disturbances in social and spiritual life, and not to psychopathological processes. Accordingly, a patient's judgement of the effectiveness of a treatment may not coincide with results on psychiatric rating scales, or, to put it in other words: therapeutic benefits may be missed when a traditional healing intervention is only evaluated by psychiatric standards [13^▪^,^17^,^28^]. Therefore, the few studies focussing on the impact of traditional healing frequently employ subjective measures such as ‘satisfaction’ or ‘helpfulness’ [17], which are summarized as ‘perceived’ effectiveness [28].

Van der Zeijst *et al.*[25^▪^] conducted a follow-up study among 42 South African apprentice traditional healers who had followed the process of ukuthwasa, the training to become a traditional healer. Nearly all individuals had completed ukuthwasa (95%) and reported a positive influence of ukuthwasa on their calling-related symptoms (98%). On the basis of thorough psychiatric assessments, the authors found a benign course of psychotic experiences and disorders after a follow-up period of 3 years, and concluded that these findings all together, suggest that ukuthwasa may be an effective, culturally sanctioned healing intervention for some selected individuals, potentially because it reframes distressing experiences into positive and highly valued experiences, reduces stigma and enhances social empowerment and identity construction [25^▪^]. Ayinde *et al.*[22^▪▪^] reported that most patients who had been treated for psychosis by TFH in Nigeria, Kenya and Ghana, were content with this treatment and would advise the same treatment to other persons suffering from psychosis. TFH were believed to provide a complete cure for psychotic illness, while treatment by biomedical practitioners was considered as ‘additional’ [22^▪▪^]. Chidarikire *et al.*[33] showed in an ethnographic study that spirituality, faith healing, religion and traditional healing played important therapeutic roles in the treatment of Shona-speaking individuals with schizophrenia in Zimbabwe, by providing a source of hope, identity, meaning and social support.

The evidence of these reviewed studies would be rated as low from a biomedical point of view, in line with most previous studies on this topic [17]. Nevertheless, they contribute to the literature suggesting that the treatment by TFH in the field of mental health in Africa, including the treatment of psychosis, is perceived to be effective by patients and their families [13^▪^,^28^].

### Harmful practices

Recent reports from Nigeria, Kenya and Ghana show that traditional and faith-based treatments of patients with psychosis might involve harmful practices such as chaining, fasting and beating [20^▪^,^22^^▪▪^,^44^^▪▪^,45^▪^]. Also, herbal concoctions and delays in start of biomedical treatment are mentioned as potentially harmful [20^▪^].

According to Ayinde *et al.*[22^▪▪^], TFH used chaining in 64% of Nigerian cases, 61% of Kenyan cases and 48% of Ghanaian cases with psychosis, and a few were locked up in rooms. These numbers from Kenya and Ghana are higher than the numbers of physical restraint reported by Esan *et al.* in 2019 [29], namely 4% of cases in Kenya and 21% in Ghana. Nevertheless, Ayinde *et al.*[22^▪▪^] reported that the restraint was often justified by patients, caregivers and TFH, as they claimed that it had been used to protect them from themselves or to protect others. Moreover, 42% of the cases stated that they had been beaten by TFH, friends, family and also biomedical health practitioners [22^▪▪^]. At a faith-based setting in Ghana, Gyimah *et al.*[45^▪^] found that the use of restraint in the form of chaining and fasting was often conducted against the will of the individuals with mental illness who were subjected to these practices. In addition, the restraint was associated with feelings of being dehumanized, emotional distress and physical pains. Despite all this, many participants justified or even valued these practices, as they believed these contributed to their healing [45^▪^].

Nyame *et al.*[20^▪^] found that Ghanaian TFH had difficulties with dealing with aggressive patients and complying to human rights due to limited resources, like the shortage of psychotropic medication at primary healthcare clinics and a lack of space to house patients. Baheretibeb *et al.*[46^▪▪^] reported that TFH from two Ethiopian faith-based settings were open to critically reflect on the need to shackle their patients. In addition, they were motivated to change ways of care and willing to learn more from biomedical health practitioners about how to manage violent behaviour of patients. At these faith-based settings, approximately 40% of the patient population is diagnosed with schizophrenia [42^▪▪^]. A study by Gureje *et al.*[44^▪▪^] in Nigeria and Ghana showed a significant reduction of harmful practices after TFH were trained and monitored to avoid harmful treatment practices among patients with psychosis. In another research setting in Ghana, solely discouraging the use of chaining did not lead to a significant reduction of days spent in chains [47].

### Collaboration

A recent study from Sudan [48] reported that most of the 108 interviewed psychiatrists and psychiatry trainees see no role for traditional healing methods in the treatment of psychosis. A systematic review by Green *et al.*[32] demonstrated that TFH and biomedical practitioners from various African countries are open to collaborate with one another in case of less severe mental disorders, despite some mutual concerns. However, current studies from Ghana, Nigeria and Ethiopia show that TFH and psychiatric care providers are also willing to cooperate in the management of patients with psychotic disorders [20^▪^,^42^^▪▪^,^44^^▪▪^]. Two of the currently reviewed articles reported that some biomedical practitioners from Ghana, Nigeria and Ethiopia were even referring patients with psychosis to a TFH [20^▪^,^22^^▪▪^].

In line with previous research on general mental health [31,32], all stakeholders report serious concerns regarding shared management of psychotic patients [20^▪^,41^▪^]. In Ghana, TFH, patients and caregivers mentioned fear of disrespect and undue criticism by biomedical practitioners as important obstacles, while primary health providers mentioned the potential harm of traditional treatments [20^▪^]. As opposed to reports on general mental health [32], a study at a holy water site in Ethiopia, where mostly psychotic patients were treated, reported that incompatibility of beliefs was an important reason for resistance among some of the holy water attendants to refer patients to the psychiatric clinic [41^▪^]. Facilitators for forging partnerships, as identified in the recent studies, were the establishment of bi-directional referrals, promoting mutual respect and recognition, handling the logistical needs of TFH in order to reduce harmful practices, training of TFHs and the provision of free antipsychotic medication [20^▪^,41^▪^,^42^^▪▪^]. In addition, the relevance of careful relationship building prior to initiating the collaboration was also stressed [41^▪^,^49^].

African examples of how formal collaborations in mental healthcare have been established and look like in practice are very limited [31,41^▪^,^42^^▪▪^,^44^^▪▪^,^50^]. Recently however, a few studies have been published, which describe actual collaborations in the management of psychosis. Gureje *et al.*[44^▪▪^] conducted a randomized controlled trial (RCT) in Nigeria and Ghana among patients with psychosis who were admitted at TFH facilities. The authors found that 6 months after the start of the study, patients who were treated in a collaborative shared care model had better symptom outcomes, less disability and less self-stigma than patients who only received care from TFH. In addition, they found that collaboration was more cost-effective [44^▪▪^]. In the collaborative shared care model, patients received care from TFH as well as from biomedical providers, who made scheduled and unscheduled visits to the TFH facilities. Another promising example of a collaboration between the treatment modalities is a project at two holy water sites in Ethiopia [41^▪^,^42^^▪▪^,^46^^▪▪^]. Since 2012, holy water attendants and priests have worked in collaboration with a psychiatric clinic to support patients with severe mental illness, mostly schizophrenia. The holy water attendants take care of the patients and bring them to holy water priests and, if they think this is necessary, to the clinic [41^▪^,^42^^▪▪^]. Baheretibeb *et al.*[42^▪▪^] reported that biomedical medication adherence was very low among 90% of the patients who made previous visits to biomedical health providers. Within the collaborative care model at the holy water sites, however, the majority of patients (92%) reported comfort in receiving psychotropic medication simultaneously with holy water treatment and prayers. Although the study lacks data in the basic clinical records to demonstrate effectiveness of the clinic, the increasing use of the clinic suggests that psychiatric care is adding value to the treatment of psychotic patients [42^▪▪^]. In a further study at the same holy water sites in Ethiopia, Baheretibeb *et al.*[46^▪▪^] developed and implemented a collaborative model that is informed by the theory of transformational learning. Transformational learning, which aims at changing perspectives, was used as a way to reconcile the two seemingly incompatible healing paradigms. The authors found that this model facilitated successful, culturally appropriate interaction between TFH and biomedical practitioners [46^▪▪^]. These case studies are examples of how the implementation of a psychiatric service near a religious healing site seems to be a practical and acceptable manner to make psychiatric mental healthcare more accessible to patients with severe mental illness, including psychotic disorders, and how collaboration between the two sectors can be enhanced [41^▪^,^42^^▪▪^,^46^^▪▪^]. Furthermore, a promising, multicentre international research project called TRANSFORM [51] is currently running in Nigeria and Bangladesh, which attempts to build an innovative and durable referral system together with TFH for individuals with psychotic disorders and severe mood disorders. The aim is to increase access to biomedical care in slums, while leveraging the advantages of already-existing pluralistic help-seeking pathways.

From these examples, it appears that completely integrated, genuinely collaborative care models, in which TFH and biomedical practitioners use a two-way referral system, are very rare in the management of patients with psychotic disorders.

## CONCLUSION

In contemporary Africa, traditional and religious beliefs dominate the views and practices regarding mental health and illness. In case of mental disturbances, including what psychiatry would understand as psychotic disorders, the majority of the population will consult a TFH as first contact or later in the process, and their interventions are perceived to be helpful. One study showed that the process of becoming a traditional healer in rural South Africa may have a positive influence on the course of psychotic symptoms in some selected individuals [25^▪^].

Many African individuals with psychotic disorders, as well as TFH, tend to have a pragmatic and integrative approach towards the understanding and treatment of psychosis, in which they increasingly seem to combine and assimilate elements of the traditional/faith-based model and the biomedical model, if they believe this to be helpful. Integration of African traditional healing into the biomedical system has been advocated for decades, especially in relation to the mental health treatment gap. Although studies have focussed mainly on the perspectives of TFH, biomedical practitioners, patients and other stakeholders towards collaboration between the two healing paradigms, there are still few – though a growing number of – examples of successful collaborations in the field of psychosis in Africa. An important challenge concerning integration/collaboration is the apparent incompatibility of and lack of respect for traditional/faith-based and biomedical frameworks, although recent studies have highlighted that there may be more common ground than previously thought. Another challenge is the lack of consensus on methods to evaluate benefits of TFH, which are acceptable to both TFH and the biomedical field. Nevertheless, the pluralistic perspectives and help-seeking behaviour of individuals with psychotic disorders suggest that it may not be necessary to harmonize both paradigms and that, despite some underlying conflicting understandings, synergistic collaboration is possible within certain limits and under certain conditions. These limits include human rights issues. In the reviewed literature, indications were found that the use of potentially harmful healing practices by TFH can probably be solved – at least for an important part – through the provision of more resources in terms of psychotropic medication and beds to admit aggressive patients, as well as through training of and collaboration with TFH. Key elements of effective collaborations are mutual respect and relationship building before the start of actual partnerships.

## Acknowledgements

*None.*

### Financial support and sponsorship

*None.*

### Conflicts of interest

*There are no conflicts of interest.*

## REFERENCES AND RECOMMENDED READING

Papers of particular interest, published within the annual period of review, have been highlighted as:

▪ of special interest
▪▪ of outstanding interest
